# Total tubeless percutaneous nephrolithotomy without artificial hydronephrosis in preschool children: Three case reports

**DOI:** 10.1097/MD.0000000000033415

**Published:** 2023-03-31

**Authors:** Xicai Zhang, Yonghe Zhang, Fengyue Li, Wenbo He, Xiande Cao

**Affiliations:** a School of Clinical Medicine of Jining Medical University, Jining, China; b Department of Urology, Affiliated Hospital of Jining Medical University, Jining, China; c Department of Pediatric Surgery, Affiliated Hospital of Jining Medical University, Jining, China; d Department of Urology, Shandong University of Traditional Chinese Medicine Affiliated Hospital, Jinan, China.

**Keywords:** percutaneous nephrolithotomy, preschool, stone, total tubeless, ureteral catheter

## Abstract

Indwelling ureteral catheter, double J tube, or nephrostomy tube are routine procedures for percutaneous nephrolithotomy (PCNL) in children. There are a few cases in which PCNL has been performed in children without any additional instruments being left in place.

**Patient concerns:**

In this study, 3 children were treated for hematuria and complicated with different degrees of urinary tract infection. All of them were diagnosed as upper urinary tract calculi by abdominal computed tomography.

**Diagnosis:**

Three preschoolers were diagnosed with upper urinary tract calculi before surgery, one with no hydronephrosis and the other 2 with different degrees of hydronephrosis.

**Interventions:**

After preoperative evaluation, all the children successfully completed PCNL without indwelling ureteral catheter, double J tube, or nephrostomy tube.

**Outcomes:**

The operation was successful and there were no residual stones observed during postoperative review. The operating times for the children were 33 minutes, 17 minutes, and 20 minutes, and the intraoperative bleeding volumes were 1 mL, 2 mL, and 2 mL. The catheter was removed on the second day after the operation, the postoperative review of the abdominal computed tomography or ultrasound did not indicate any stone residue, and there were no fever, bleeding, and other related complications after the operation.

**Lessons:**

We believe that total tubeless PCNL without artificial hydronephrosis can be achieved in the preschool population.

## 1. Introduction

In recent years, there has been an increase in the incidence of stones in children, and percutaneous nephrolithotomy (PCNL) has been widely used to remove kidney stones in children because of its high stone removal rate.^[1]^ The placement of a ureteral catheter through a cystoscope, postoperative indwelling ureteral stent, and nephrostomy tube are the routine steps of percutaneous nephrolithotomy, which is mainly aimed at dilating the collecting system to improve the success rate of puncture, postoperative drainage, and compression to stop the bleeding. However, the urethra of preschool children is thin and curved, and leaving or removing ureteral catheters and ureteral stents increases the risk of damaging the urethra or ureteral mucosa, which can lead to postoperative urethral stricture and irreparable damage to the child. We report the clinical experience of 3 cases of PCNL without an indwelling ureteral catheter, ureteral stent, and nephrostomy tube for the treatment of stones in preschool children.

## 2. Illustrative cases

### 2.1. Case1

**Figure 1.**
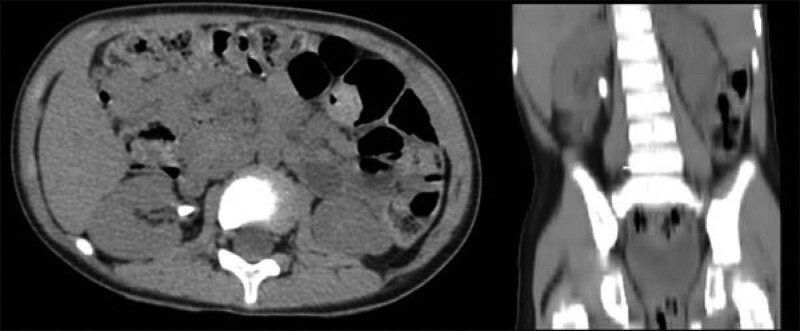
Sagittal and coronal view of lower abdominal CT before operation (Case 1). CT = computed tomography.

A 3-year-old boy had a right renal stone for more than 3 months with carnivorous hematuria. Urine leukocyte count was 12.36/µL and urine culture results were negative. Preoperative abdomen computed tomography (CT) indicated a stone in the right renal pelvis-ureteral migration with a stone size of about 1.2 cm (Fig. 1). Ceftriaxone sodium was given before the operation and the operation was performed on the second day of admission.

### 2.2. Case2

**Figure 2.**
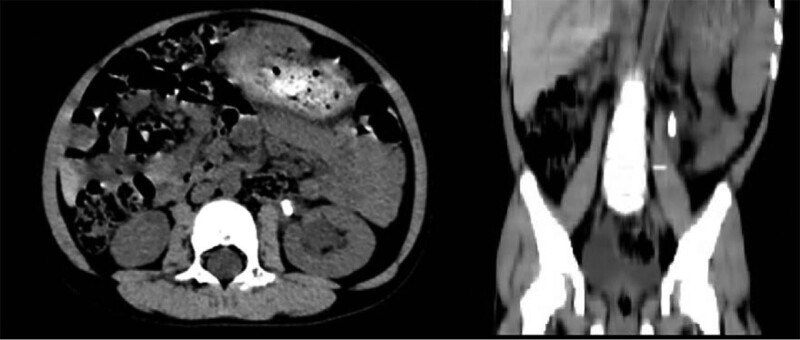
Sagittal and coronal view of lower abdominal CT before operation (Case 2). CT = computed tomography.

A 3-year-old boy had been admitted to the hospital for 3 days with hematuria. Urine leukocyte count was 64.02/µL, urine culture results were negative, and preoperative abdominal CT indicated a stone in the right renal pelvis-ureteral migration with a stone size of about 1.8 cm (Fig. 2). Ceftriaxone sodium was given to prevent infection before surgery. The operation was performed on the third day of admission.

### 2.3. Case3

**Figure 3.**
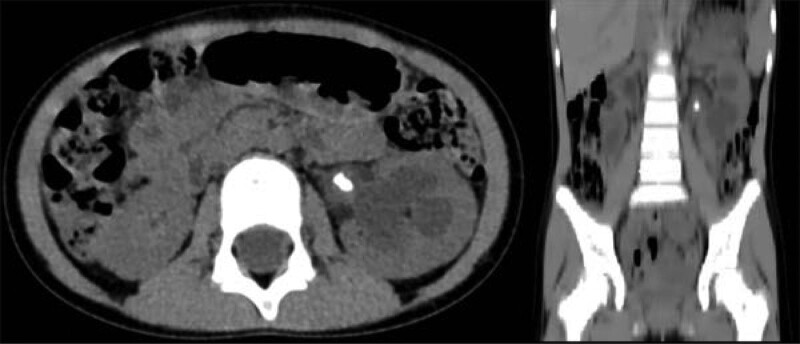
Sagittal and coronal view of lower abdominal CT before operation (Case 3). CT = computed tomography.

A 4-year-old girl had been admitted to the hospital for 2 days with hematuria. Urine leukocyte count was 18.26/µL and urine culture results were negative. Preoperative abdominal CT showed that there were one calculus at the ureteropelvic junction and one in the lower ureter, with sizes of 0.6 cm and 0.3 cm, respectively (Fig. 3). Preoperative anti-infection treatment with ceftriaxone sodium was given for urinary tract infection and surgery was performed after infection control. The upper ureteral calculus was removed using percutaneous nephrolithotomy, and the lower ureteral calculus was removed using ureteroscopic lithotripsy.

## 3. Surgical procedure

**Figure 4.**
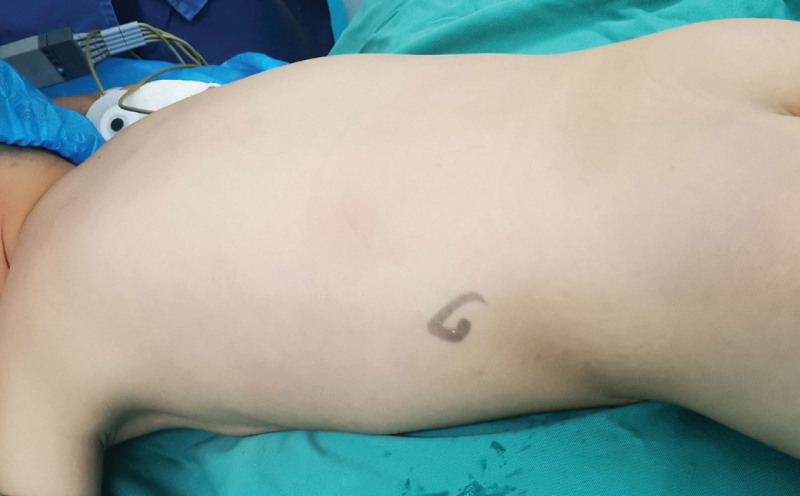
Picture of the preoperative body position.

**Figure 5.**
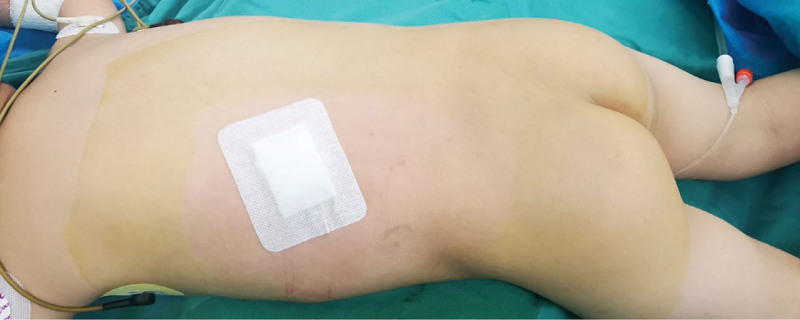
Picture of the end of the operation.

The child was anesthetized by static suction compound anesthesia, an F6 silicone catheter was placed, and the skin puncture site was selected from the subscapular angle line to the posterior axillary line in the 11th intercostal or 12th subcostal margin in the prone position (Fig. 4). Based on the preoperative CT and the intraoperative ultrasound exploration, the target renal calyces were selected and the anterior end of the 18G puncture needle was successfully inserted into the collecting system. The needle core was withdrawn and urine flow was observed, and subsequently a 0.038-inch zebra guidewire was introduced and the skin was incised at the puncture site for approximately 0.8 cm. A 16F fascial dilator was then introduced to establish channel by one-shot dilation, the nephroscope was inserted, and the Holmium laser was used to break up the stone. The remaining renal calyces were inspected for residual stones before the nephroscope was withdrawn and the incision was sutured. No double J tube and nephrostomy tube were left in place in children (Fig. 5), and all procedures were performed by the same operator.

## 4. Results

**Figure 6.**
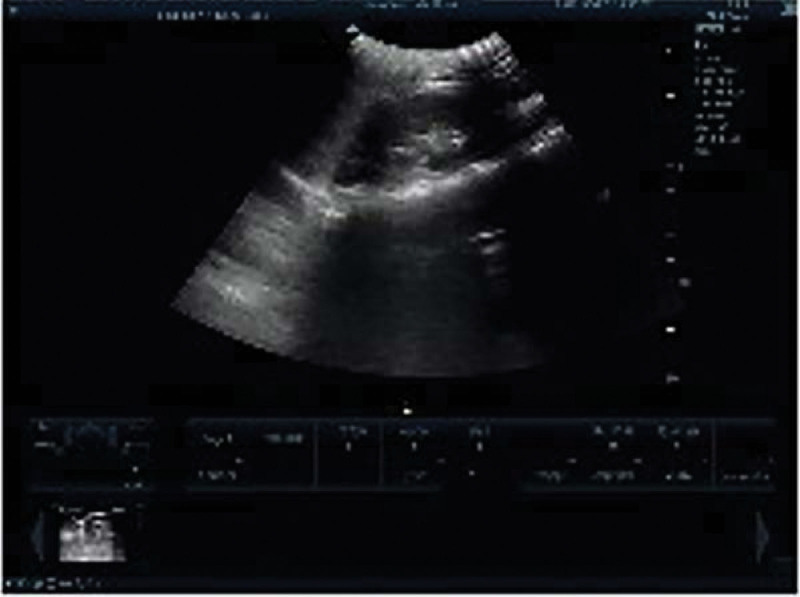
Postoperative ultrasonography (Case 1).

**Figure 7.**
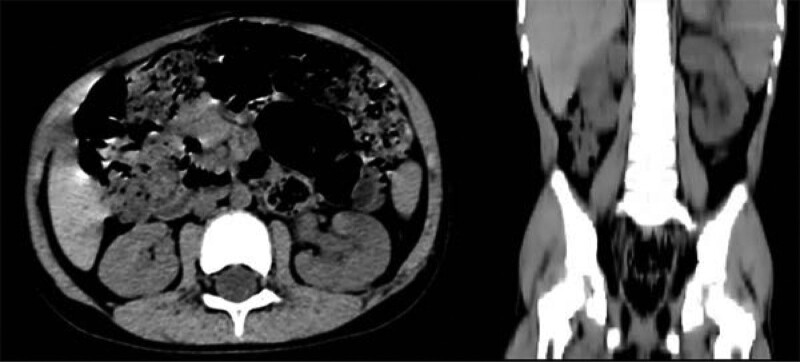
Sagittal and coronal view of postoperative lower abdominal CT (Case 2). CT = computed tomography.

**Figure 8.**
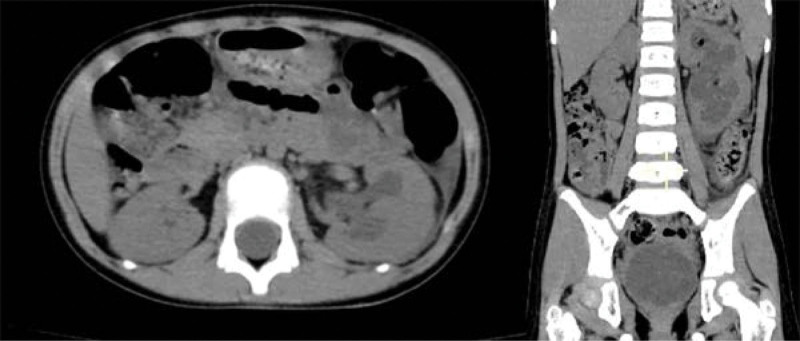
Sagittal and coronal view of postoperative lower abdominal CT (Case 3). CT = computed tomography.

The operation was successful and there were no residual stones observed during postoperative review (Fig. 6–8). The operating times for the 3 children were 33 minutes, 17 minutes, and 20 minutes, and the intraoperative bleeding volumes were 1 mL, 2 mL, and 2 mL. The catheter was removed on the second day after the operation and the postoperative review of the abdominal CT or ultrasound did not indicate any stone residue. Furthermore, there were no fever, bleeding, and other related complications after the operation. The postoperative hospital stay durations were 2 days, 3 days, and 3 days (Table 1).

**Table 1:** Demographics and calculi-related characteristics of patients.

Items	Case1	Case2	Case3
Age (yr)	3	3	4
Gender	Male	Male	Female
Multiple stones length diameter (cm)	1.2	1.8	0.9
Intraoperative bleeding (mL)	1	2	2
Operative time (min)	33	17	20
Postoperative hospitalization time (d)	2	3	3
Single-stage stone free rate (%)	100%	100%	100%

## 5. Discussion

At present, PCNL is one of the main procedures for the treatment of children with complex stones. Based on the small and unique anatomical features of children, PCNL has been gradually developed in the direction of refining the instruments and simplifying surgical operations. Our center has discussed the application of PCNL without a ureteral catheter, ureteral stent, and nephrostomy tube in preschool children.

Safe and successful puncture and establishment of access is one of the key steps in determining the success of PCNL surgery,^[2]^ and this is especially true for pediatric PCNL because of the small anatomy of the child. In current studies on PCNL for the treatment of pediatric stones, intraoperative ureteral catheters need to be left in place, through which saline can be injected to establish an artificial hydronephrosis dilated collecting system, which can reveal the dome of the target calyces and improve the success rate of the puncture, especially for patients with stones without hydronephrosis.^[3]^In addition, it has the effect of preventing the downward migration of stones to avoid a secondary ureteral extraction. However, there are several disadvantages to this operation; If the child has a twisted ureter or ureteral stenosis, it will lead to the failure of ureteral catheterization; The immune system of the child is not sound, and the high pressure of the retrograde saline infusion will easily lead to bacterial re-infiltration into the blood, increasing the incidence of postoperative infection^[4]^; Retrograde infusion of saline or contrast agent will lead to extravasation of fluid or contrast agent,^[5]^ which in turn can affect their imaging under ultrasound; The child’s position needs to be changed twice during the procedure, which prolongs the operation time and increases the risk of infection; and The anatomical features of the child are smaller, and the urethra and ureter are thinner and more curved than those of adults, which increases the risk of damaging the ureter and urethra, resulting in postoperative ureteral stricture. In order to avoid such risks, no ureteral catheter was left in place in the 3 children in this study, and all of them were successfully punctured in a single attempt, without reducing the success rate of the puncture or increasing the bleeding-related complications. The average operation time was 23.3 minutes, which was significantly shorter than the operation time in other studies^[3]^ and reduced the risk of anesthesia. In this study, there were no secondary stone extraction requirements related to the downward migration of stones.

The current study concluded that ureteral stents or nephrostomy tubes need to be left in place after PCNL surgery for drainage and to avoid postoperative infection, but they need to be removed twice after surgery, which increases the child’s discomfort. Especially for the ureteral stents where the child cannot tolerate cystoscopy, to the stent needs to be removed through cystoscopy under anesthesia, which increases the risk of anesthesia and prolongs the hospital stay. In this study, all 3 children did not have an indwelling nephrostomy tube and ureteral stent, which reduced their discomfort. The findings by Goktug HN et al^[6]^ support these results.

## 6. Conclusion

In this study, no ureteral catheter, ureteral stent, and nephrostomy tube were left in place during the PCNL procedure, which simplified the procedure and allowed a successful puncture in a single attempt, thus minimizing the damage to the child. Therefore, we believe that total tubeless PCNL without artificial hydronephrosis can be achieved in the preschool population.

## Author contributions

**Conceptualization:** Yonghe Zhang, Xiande Cao.

**Data curation:** Xicai Zhang, Xiande Cao.

**Funding acquisition:** Xiande Cao.

**Methodology:** Fengyue Li.

**Writing – original draft:** Xicai Zhang, Xiande Cao.

**Writing – review & editing:** Wenbo He, Xiande Cao.
